# Developing Nepal’s medicines pricing policy: evidence synthesis and stakeholders’ consultation

**DOI:** 10.1080/20523211.2024.2346222

**Published:** 2024-04-29

**Authors:** Zaheer-Ud-Din Babar, Santosh Dulal, Narayan Prasad Dhakal, Madan Kumar Upadhyaya, Birna Trap

**Affiliations:** a Department of Pharmacy, University of Huddersfield, Huddersfield, UK; b USAID Medicines, Technologies, and Pharmaceutical Services (MTaPS) Program, Management Sciences for Health, Kathmandu, Nepal; c Department of Drug Administration, Ministry of Health and Population, Kathmandu, Nepal; d Quality Standard and Regulation Division, Ministry of Health and Population, Kathmandu, Nepal

## Abstract

**Objectives:**

The objectives of this paper are to (a) explore stakeholders’ opinions regarding Nepal’s existing medicines pricing practices/situation and (b) build and present a set of medicines pricing policies for Nepal.

**Methods:**

A review of the literature and field visits to community retail pharmacies, hospital pharmacies, wholesalers, and distributor outlets in Kathmandu were conducted to assess the medicines pricing situation. Following the literature review, preliminary meetings with stakeholders and field visits were held and a draft interview guide was prepared. Consultative sessions subsequently were undertaken in Kathmandu, Nepal, in January 2023 with representatives from the Department of Drug Administration, Ministry of Health and Population, Association of Pharmaceutical Producers of Nepal, consumer groups, Transparency International, Medicines Importers Association of Nepal/ Pharmaceutical Distributors Association of Nepal, Nepal Chemist and Druggist Association, and Nepal Pharmaceutical Association. Notes were taken during these meetings regarding issues and concerns raised as well as experiences and recommendations for the future, as outlined in the interview guide.

**Results:**

The stakeholders in general stated that they do not have any objection to price regulation; however, they believe such regulation should be subject to periodic review. Both the importers and the Ministry of Health and Population have the view that an independent body/authority should be charged with regulating the prices of medicines. A set of policy options to be considered for use in Nepal include cost-plus pricing, external price referencing, internal reference pricing, and mark-up regulations.

**Conclusion:**

Key issues related to pricing were identified and suggest that a set of pricing policies and updated regulations need to be considered to establish changes that are transparent, rational, and acceptable to the related stakeholders. Hence, suggestions made in this paper could be useful to inform a rational and fair pricing structure and to improve access to medicines.

## Introduction

In 2019, Nepal spent USD 53 per capita on health. Like in many other low- and middle-income countries (LMICs), out-of-pocket expenditures in Nepal are very high, and about 56% of health expenditures are in the private sector. (World Data Atlas; World Bank Data) Nepal has approximately 169 importers, 128 domestic manufacturers, 17,000 types of medicine (half of which are manufactured domestically), 3,400 wholesalers, and 28,000 pharmacies. (Drug Bulletin of Nepal, 2019)

### Medicines pricing in Nepal

The government of Nepal established the Department of Drug Administration (DDA) in 1979 to ensure safety, efficacy quality and rational use of medicines in the country. The DDA was given the mandate to regulate and control the production, marketing, distribution, export-import, storage, sale, distribution, and utilisation of medicines.

In 1995 the medicines pricing committee regulated the price of dextrose followed in 2007/8, by DDA setting the maximum retail price (MRP) for other selected medicines by exercising the power conferred to it by section 26 of the Drug Act 1978. This arrangement is similar to that used in other countries in South Asia, including India, Pakistan, Bangladesh, and Sri Lanka, (Babar, 2015; Babar, 2022) where MRP is an ex-factory price that is the highest price that can be charged for a medicine sold at a retail pharmacy.

However, Nepal’s medicines pricing policy and regulation is somewhat different from some other regulations because there is no legal framework or mechanism in place to adjust the MRP over time and it does not include standard criteria to set the MRP of an individual medicine. (Hogerzeil et al., 2022)

In Nepal, the DDA has stated that the MRP of an imported medicine cannot be higher than the MRP for that medicine in the exporting country. This could have implications, because if the price of an imported medicine increases and the Nepal MRP is not adjusted, there is a change that medicine may be withdrawn from the market. (Hogerzeil et al., 2022) A similar situation could occur if the price of raw materials used to manufacture a medication domestically increases with no mechanism for adjusting the MRP to account for that increase.

Currently, the MRP for 117 molecules of medicines of the same dosage form and strength is fixed under section 26 of the Drug Act 1978. (Devkota et al., 2018) However, some of the prices that were set in 2007-2015 have not been adjusted since. Also, no established mechanism to continuously track adherence to the designated price over time exists.

In Nepal, distributors and wholesalers sometimes might sell medicines directly to the hospital or the patient such as in the case of the sale of some expensive cancer medicines. In case of direct delivery by distributors to the patient, there is no mechanism in place to assess adherence to MRP and control mark-ups at different levels of the supply chain. In the private sector, the profit margins for some medicines can be up to 33% by the time those medicines reach consumers. (Hogerzeil et al., 2022) In addition, often times, the profit margins for retailers (16%) are in addition to discounts and bonuses provided by the manufacturer and or distributors. These bonuses and discounts creating financial [open-strick]dis[close-strick]incentives for retailers and middlemen however its costing rational and cost-effective prescribing. (Hogerzeil et al., 2022)

### Literature synthesis and the rationale of the study

A literature review was conducted to assess the medicines pricing situation in Nepal. Scientific databases were searched, and 13 studies were found on ‘Nepal medicine pricing.’ Those studies reported a wide variation in the prices of medicines in the country, (Devkota et al., 2018; Poudel et al., 2018) with the prices of the same medicines and formulations from different manufacturers varying greatly.

A DDA study conducted in 2006 evaluated the price variations among Nepalese brands for different medicines. Analyzing a total of 79 drugs and 537 brands, the study found that the greatest price variation in MRP was found in the group of anthelmintics, followed by antihistamines and antiemetics. Among the individual drugs, fexofenadine and albendazole showed the highest price variations. Variations were also observed in the prices of different brands of medicines with the same active ingredient being manufactured by different Nepalese companies. (Shankar et al., 2006)

Studies have also revealed substantial price variations in medicines used for the management and treatment of non-communicable diseases (NCDs) (Devkota et al., 2018; Poudel et al., 2018). A cross-sectional study was carried out in 2017 of 94 community pharmacies of Kathmandu Valley. The study showed high competition and high price variation in the case of metformin 500 mg (255%) and atorvastatin 10 mg (328%) among different manufacturers and between locally produced and imported medicines. The study also showed access to cost-effective brands was poor (Shrestha et al., 2017).

One study also evaluated the price variations for medicines of different brands used to treat chronic obstructive pulmonary disease, cardiovascular disease, and diabetes. Among the 46 medicines studied, 32 (70%) displayed price variation percentages that ranged from 0.5% to 60%. The prices of two different brands of the same generic medicine were different even though the prices were fixed by the government (Poudel et al., 2018).

The price variations for antihypertensive drugs, cardiovascular medicines, and oral hypoglycemic drugs were also noted in several other studies (Karki et al., 2021; Khanal et al., 2019; Mishra et al., 2015; Sharma et al., 2021; Shrestha, 2015). The average percentage price variation for different brands of the same oral hypoglycemic drugs manufactured in Nepal is wide (Shrestha, 2015). A wide cost ratio (CR) and cost variation (CV) were also observed among antihypertensive medications, including amlodipine 5 mg (CR: 3; CV: 233%) and Losartan 50 mg (CR: 32; CV: 3,132%) (Karki et al., 2021). However, price variations were not limited to cardiovascular or diabetes medicines but were also reported for other classes of medicines. A study (Shrestha et al., 2020) conducted to assess price variation for 31 anticancer medicines suggested the need to implement an inclusive price control policy with a primary focus on essential medicines, without compromising the quality and availability of all medicines (Poudel et al., 2020).

Though Nepal has a drug policy from 1995 that include the objectives of ensuring affordable medicines and develop suitable mechanisms to ensure reasonable prices, the lack of an official medicines pricing policy for Nepal is having far-reaching impacts on the pharmaceutical industry, pharmaceutical supply chain, retailers, prescribers, and consumers. The supply chain is not transparent about profit margins, add-ons, and mark-ups, which is also having an impact on prescribers and pharmacists as bonuses on drugs could incentivize the irrational use[open-strick]d[close-strick] of medicines. Rational medicines pricing will not only allow transparency along the supply chain but will also improve medicines equity and access for the masses. A structure on fair pricing of medicines therefore is needed. This study was designed and conducted in that context.

It was on that background that the Ministry of Health and Population (MOHP), led by the DDA with support from the World Health Organization (WHO) and the US Agency for International Development Medicines, Technologies, and Pharmaceutical Services (MTaPS) Programme, initiated the process of reviewing the current pricing situation for pharmaceutical products in Nepal, describing international practices on pharmaceutical products pricing, and outlining a road map for the formulation of price regulation of pharmaceutical products in Nepal (USAID Medicines).

### Objectives of the study

The study objectives were to (1) evaluate Nepal’s medicines pricing situation and (2) recommend a set of pharmaceutical pricing policies for the country.

## Methodology

To undertake a situation analysis, several meetings were initially arranged with key officials from the MOHP, the DDA, and the Nepal Medical Council to align international best practices with Nepal’s legislation, law, policy, and guidelines. Two field visits were conducted to five community retail pharmacies, three hospital pharmacies, three wholesalers, and five distributor outlets in Kathmandu. Based on the preliminary meetings with the key officials, literature review, and field visits, a draft interview guide was prepared to document the medicines pricing situation as well as to seek suggestions during a consultative session with key stakeholders.

The meetings and consultative sessions were undertaken with key stakeholders, including representatives from the DDA, MOHP, Association of Pharmaceutical Producers of Nepal (APPON), consumer groups, Transparency International, Medicine Importers Association of Nepal (MIAN)/Pharmaceutical Distributors Association of Nepal (PDAN), Nepal Chemist and Druggist Association (NCDA), and Nepal Pharmaceutical Association. Each consultative session lasted approximately half a day, and notes were taken. These sessions were conducted in Kathmandu in January 2023 in collaboration with the DDA. The meetings were led by the DDA, MTaPS, and the international advisor.

Each consultative session started with a recitation of the aims and objectives, followed by a presentation on global and local pharmaceutical pricing, findings from the literature review, and documented issues with existing price regulation. The presentations were followed by a discussion driven by the interview guidelines, including key questions related to pricing, regulation, and their impact on consumers that were developed from the detailed literature review. Key questions related to price fixing policy as well as current practices of medicine pricing in Nepal were put on the table. Major challenges associated with medicines pricing and barriers, as well as solutions for forming a medicines pricing policy, were also discussed. Some questions addressed the role the DDA should play in price regulation, authority that should be responsible for regulating the prices of medicines, and the legal basis for fair pricing regulation.

The challenges faced by the DDA in regulating the prices of medicine were also explored. Other key questions addressed international practices in price regulation and their applicability in the context of Nepal. Current practices in the medicines supply chain, including gift, bonus, free goods and deals, franchises offered to retailers and other influences on prescribers for promotion of pharmaceutical products as well as possible control mechanisms, were also explored.

The study focused on stakeholders’ views of existing challenges. It did not discuss or address possible solutions with stakeholders, whose knowledge of alternative solutions was limited.

Appendix 1 describes the list of abbreviations used in the study.

## Results

The key findings and thematic analysis from the consultative stakeholder meetings are presented below.

### MOHP and DDA

Financial security and health resources mobilisation are critical components of universal health coverage, which must be equitable in order to safeguard those who are least able to pay. Pharmaceutical expenditures are a significant driver of increasing health care costs, especially in countries such as Nepal, where out-of-pocket expenditures are common. High out-of-pocket expenditures have been addressed in Nepal through price regulation and the updated draft National Medicines Policy, as well as through the introduction of a health insurance scheme ensuring that a package of 98 essential medicines is available free of charge in the public sector.

However, the challenges are many, and the need to update the existing medicines price regulation and establish a mechanism for updating MRPs is well recognised. The DDA is facing an overall shortage of skilled staff, especially qualified professionals in the field of economy and medicines pricing. A separate entity is needed for medicines pricing regulation, coordination, and communication situated i.e. at the DDA or the MOHP. . Moreover, Nepal needs to align its legislation/laws, policy, and guidelines with international best practices. An autonomous body that includes economists, finance experts, and experts from related fields, as well as a framework for fair price regulation duly approved by the cabinet is needed.

### Medicine importers association of Nepal/pharmaceutical distributors association of Nepal

MIAN and PDAN stated that they have no objection to price regulation but believe any such regulation should be subject to periodic review, noting that some medicines have had the same fixed prices for the last 10–15 years. They stated that price regulation should be the responsibility of an independent body under the prime minister's office. The current average wholesaler margin in multinational or transnational (not local) companies is 7%, whereas the margins for franchise marketing companies are up to 20%. Indian non-branded generic items are cheaper in Nepal. They noted that bonuses of up to 300% are available on some items, e.g. pantoprazole, with manufacturers competing to provide bonuses and gifts. For the benefit of the general public (consumer/patient), manufacturers, importers, wholesalers, and retailers, these unfair strategies need to be eliminated and a fair and transparent margin (benefit) provided to each specific stakeholder.

### Nepal pharmaceutical association

The Nepal Pharmaceutical Association sees a need for price regulation and an independent body established under the MOHP to be responsible for that regulation. The Association said price control could result in issues linked to availability – for example, strict price control might make sustaining a business in the retail pharmacy sector challenging due to decreased profitability – and may also impact the quality of medicines. The Association’s representative said the possible negative impact of price regulation should be analysed and expensive items such as human albumin, which costs Rs. 2000 in India but Rs. 5000 in Nepal, should be regulated first.

The raw materials suppliers for the local manufacturer are the same as those that supply manufacturers in India, but the price of the same generic is different for local and nonlocal manufacturers. The Nepal Pharmaceutical Association said the profit margin for community pharmacies should be 20% or more but suggested that a price ceiling may not be workable. The Association said price regulation should address pricing by both local manufacturers and importers, noting that the same generic with similar packaging may exhibit huge price differences.

Price control should start with the vital (essential) drugs, the Association said, and a survey should be conducted about the bonuses and incentives provided by companies to prescribers and retailers before establishing a price ceiling. There should be a price differential between common/’me-too’ products, slow-moving/rare products, and expensive items.

### Consumer forums and transparency international

The consumers said that an independent body/authority should be established to regulate the prices of not only medicines and health technological products but also other items. The DDA can also work on prices, but an autonomous body is preferred. The DDA should not take on responsibility for price regulation and managing pricing policy in Nepal but instead should focus on ensuring and regulating the safety, efficacy, and quality of medicines. The consumers’ representative said an authority could be established through an act, price regulation should be transparent, and the pricing authority should also perform the functions of market price monitoring and evaluation.

A transparent price setting and changing formula should be established, and all stakeholders should understand the fair pricing mechanisms. Noting that medicines nearing their expiry date are being sold at cheaper prices, the consumers said this aspect also needs to be regulated, as do cosmeceuticals and nutraceuticals, which are being sold at very high prices. Strong political commitment is required for the implementation, and proper monitoring of that implementation is a must. The task of monitoring for compliance with fixed MRPs can also be given to the local government. Certain loopholes in Nepal’s Drug Act 1978 and legal documents need to be addressed, according to the consumers. The DDA may evaluate medicines and deregister items that are found unnecessary to decrease the number of items in the market. The ethical and moral responsibility of each stakeholder for the fair and affordable price of medicine should be clearly outlined, and the concerned stakeholders should be held socially, legally, and ethically responsible.

### Association of pharmaceutical producers of Nepal

APPON is not against pharmaceutical price regulation but believes it should be undertaken by an independent authority – not the DDA or MOHP. In its present form and structure, the DDA cannot regulate prices due to inadequate human resources and a lack of infrastructure. The DDA should focus on the quality of medicines and other governance issues. Having the quality and prices regulated by the same organisation might impose various challenges. There should be a robust system to review the prices of medicines. Stakeholders’ involvement and multisectoral participation are needed. When the DDA started fixing prices more than two decades ago, the public initially recognised its usefulness. However, the fixed prices of several medicines have not been revised since then, which has created a problem regarding their availability. APPON has been demanding that the prices of the medicines on which the price ceiling is made be reviewed, but the government has not acted on the Association’s demand to revise the current ceiling. One method for medicines price regulation is the cost-plus method, which is not feasible for Nepal, according to APPON. The best option for price control is to use an external reference price as a benchmark. The authority should be equipped with experts and resources and should be a functional board that regularly revises the prices of medicines. An efficient information technology system should be used for the automatic fixing of prices. APPON is advocating for a long-term solution or to build a fair pricing policy for medicines. APPON is very aware of its responsibilities. The focus should be on adopting a fair and transparent pricing policy.

### Nepal chemist and druggist association

The NCDA stated that price fixing is not the main issue of concern for NCDA members (retailers, distributors, and suppliers). The NCDA highlighted that the manufacturers are giving benefits to doctors (prescribers), retailers, and government offices. Also, local manufacturers often do not have adequate research and development (R&D) costs compared to foreign manufacturers.

Medicines pricing in Nepal is based on external reference prices. Around 50% of medicines in Nepal are imported from other countries, primarily India, and the labels of those products are imprinted with the MRP from the country of origin. The imported products are not relabelled; instead, they are sold with the foreign product label intact, and the MRP is converted into the Nepalese currency at the point of sale to the consumer. Domestic companies also mentioned their MRPs referencing Indian prices, stating that a price ceiling could have a negative impact, as several manufacturing companies producing infusions have closed because of low MRPs.

To regulate the prices of medicines, the DDA established a Drug Price Monitoring Committee tasked with setting the price for 96 essential drugs. The NCDA is a member of that committee; however, the committee is currently inactive. The NCDA stated that it has no objection to price regulation as long as it is dynamic and undergoes periodic review by an authority independent of the DDA. Such an autonomous body should include market experts, as well as pharmaco-economists, market economists, and other key experts and stakeholders. Reference pricing could be used for setting the price. Medications should be prescribed using their international non-proprietary names (INN), and generic prescribing should be implemented.

Companies that do not have R&D facilities and/or do not adhere to good manufacturing practices (GMP) charge the same price as those that do, which should be addressed. The current practices in Nepal does not focus on optimising and fully utilising available capacity and the regulatory system of Nepal does not allow the sharing of plant machinery among manufacturers, but sharing provisions would allow the machines to function at full capacity, which would reduce the price of medicines, the NCDA said.

## Discussion

Price regulation is important for Nepal to ensure access to effective, safe, quality assured, and affordable medicines for all and to establish fair medicines pricing mechanisms that can improve access, ensure affordability, and support the pharmaceutical industry. However, understanding the global medicines pricing setup is vital. The major takeaway from the stakeholder consultations was that prices should be regularly updated and ensured to be fair and that regulations should be established by an autonomous, well-resourced body in a transparent manner. Global experiences and practices with price regulations should be explored to determine how regulation can be optimised for implementation in Nepal. Nepal can learn from international practices and search for the best options for the country. The main implications, as well as the pro and cons of various price regulation principles, are discussed below.

### An autonomous medicines pricing regulatory authority and fixing of fair prices

There is evidence from LMICs, (Schneider & Vogler, 2016) as well from as high-income countries (e.g. Germany Glaeske, 2008), that limited price regulation can lead to high prices. No clear relationship has been shown between a country’s income and its medicine price level (Vogler, 2022). Nevertheless, weighted for purchasing power parity, some low-income countries may be paying higher prices (Moye-Holz & Vogler, 2022).

Fixing prices of medicines is a challenging issue – a point that was raised by various stakeholders. The prices of some medicines, including IV fluids, were fixed in 1995 and last reviewed in 2007 and presently over900 molecules need to have the prices fixed.. Nearly all stakeholders agreed that an independent authority is needed to regulate the prices of medicines. There was no objection to the price regulation so long as periodic reviews are place.

A pricing authority can be created either within the MOHP (independent of the DDA) or as an independent entity under the government. However, as stressed by stakeholders, the authority should be well resourced, feature sufficient expertise and representation, and be autonomous to make independent, necessary recommendations and regulations. The autonomous authority can implement and enforce the provisions of the drug pricing regulations.

The systems or concepts for medicines pricing that might be applied were discussed and mainly giving priority to an external reference pricing or a cost-plus method.

The most difficult task is establishing reasonable, fair, and appropriate prices for Nepal (Rietveld & Haaijer-Ruskamp, 2002). Countries differ in their definition of a reasonable maximum price, depending on such factors as budget limits, prescribing behaviour, demographics, and the strategic importance of the pharmaceutical industry to the national economy (Mossialos et al., 2006). Techniques used to calculate reasonable maximum prices also vary. Combinations of two or more of the following techniques are most often used globally: external reference pricing, internal reference pricing, cost-plus pricing, and profit ceilings (Jacobzone, 2000; OECD, 2008; Productivity Commission, 2001; Rietveld & Haaijer-Ruskamp, 2002; Vogler et al., 2008).

Benchmark prices along with the other South Asian Association for Regional Cooperation (SAARC) countries was also suggested. However, in many SAARC countries, prices are not updated and discounts and rebates are not disclosed. Understanding of the economy of how the market functions is needed to apply suitable measures to control prices.

It was also stated that controlling the MRP alone will not work – the entire pharmaceutical supply chain needs to be regulated, including manufacturers and importers. Medicine availability issues may arise in the wake of price control, and the sustainability of retail pharmacies in a price-controlled environment remains is a question. In Nepal, it was also suggested that the profit margin of community pharmacies should be at least 20%. Price control should begin with national essential medicines, but cosmeceuticals and nutraceuticals are being sold at very high prices and should also be regulated.

#### Issues with the pharmaceutical industry

The pharmaceutical industry needs to be supported. Nepal has a window of opportunity under the World Trade Organisation Agreement on Trade-Related Aspects of Intellectual Property Rights (TRIPS) waiver in effect until 2033; hence, an opportunity exists to produce generic copies of innovative medicines. TRIPS is the most comprehensive multilateral agreement on intellectual property. Taking advantage of this opportunity will allow for more affordable medicines to become available in Nepal.

#### Import of medicines

A large majority of medicines that are imported into Nepal come from India. Indian MRP currently is used to set the price of a medicine in Nepal, but it is important to note that the one Indian Rupee is equivalent to 1.6 Nepalese Rupee. Moreover, there are eight border points and an open border between Nepal and India, which facilitates the entry of medicines into Nepal from India. Regulation of prices for products that are imported into Nepal, especially from India, must be such that both locally produced products can be competitive and illegal cross border trade is not incentivized.

#### Rational prescribing, unethical medicines promotion, and bonuses, rebates, and profit margins

Rational prescribing should also be linked to price regulation. Surveys and research should be conducted regarding the bonuses and incentives provided to prescribers and retailers before setting a price benchmark. Bonuses and rebates are given to prescribers and community pharmacists, and, in some instances, to wholesalers and distributors if they sell more medicines.

Bonuses and incentives should be abolished through regulation – there should be no bonus system for either locally produced or imported products. To promote rational prescribing, physicians should prescribe with INNs.

#### Prices of various products

The questions of whether the prices of certain medicines should be fixed and whether price fixing should be only for essential medicines were also discussed. Also discussed was whether a special mechanism is needed to set the prices of anticancer medicines, high-cost medicines, hepatitis C treatment, remdesivir, etc. Most stakeholders stated that the prices of all medicines should be fixed – not only essential medicines and supplies. However, most agreed that regulating all medicines is a huge undertaking and the starting point should be essential medicines and supplies.

#### Drug shortages

Shortages of several medications, notably including verapamil, sodium bicarbonate, potassium carbonate, and 50% dextrose, are reported in Nepal. Although some have linked those shortages to pricing issues, whether that is accurate needs to be investigated further.

#### Quality of medicines

Many question the quality of medicines in Nepal, and the issue came up quite often during pricing discussions. Participants stated that pharmaceutical companies without R&D facilities and that do not adhere to GMP are charging the same prices as are those that are GMP compliant and having R&D expenses. However, it is important to note that only medicines meeting GMP requirements should be registered with Nepal’s DDA regardless of price regulation, and post market surveillance with regular checks of the quality of medicine should be implemented. According to best international practices, pricing should never be related to the quality of medicines.

#### Price cut or price freeze

After market entry, some countries allow inflationary price increases; however, others limit price increases, enforce a price freeze, or instigate price cuts over time. In Switzerland and Vietnam, price increases are permitted post-entry, but pharmaceutical companies are required to file an application providing a rationale for the increases (Critchley, 2006; Espin et al., 2011; GO G/O BIG, 2006; Leopold et al., 2012; Nguyen, 2011; Nguyen et al., 2014; OECD, 2008; Vogler et al., 2008).

In Canada and Hungary, any price increases are limited to the rate of inflation, (Critchley, 2006; Nguyen et al., 2014) while Sweden and the Slovak Republic do not permit price increases, except under exceptional circumstances (Vogler et al., 2008). The UK launched a price freeze for generic medicines in 1999, and Ireland implemented a price freeze agreement in 2006 (Vogler et al., 2008). Germany, on occasion, has imposed price freezes and demanded across-the-board rebates to tackle deficits in health insurance funds (Paris & Docteur, 2008). Price cuts or price freezes often occur following a price review by the government. Some countries also use margin cuts to limit the profits of distributors (Vogler et al., 2008). Challenges in several Asian countries (such as Pakistan, Bangladesh, and India) relate to a lack of reliable data on medicines pricing and the mechanisms of price regulation.

#### Percentage and mark-ups – global examples

The Federation of Bosnia and Herzegovina introduced medicines price regulation based on international reference pricing, setting a maximum allowed wholesaler price using Slovenia, Croatia, and Serbia as reference countries. This resulted in a price decrease. The wholesale margin in Bosnia and Herzegovina is regulated and set at 8%, while in the Republic of Srpska it is 6%. The retail pharmacy margin in Bosnia and Herzegovina is defined up to 25%, while in the Republic of Srpska it is 18% (Izham et al., 2022). Some other global practices that can be followed are to set the domestic retail price of an innovator product equal to the lowest retail price available worldwide for the same product (Espin et al., 2011).

### Setting up a medicines pricing policy for Nepal

Several different approaches can be taken with respect to setting up a medicines pricing policy that achieves fair prices while improving affordability for consumers. Among the approaches recommended by the WHO (WHO guideline on country pharmaceutical pricing policies; World Health Organization’s Regional Office for South-East Asia (SEARO); World Health Organization) are internal reference pricing, cost-plus pricing, external reference pricing, value-based pricing, and mark-up regulations.

#### Pricing options

##### Cost-plus pricing policy

One of the suggested options for Nepal is a cost-plus pricing policy (see Table 1 for details).

**Table 1.** Pharmaceutical pricing policies for Nepal.

Pricing Policies	Global Practices	Challenges	Application/Mark-ups/percentage	Applying at what level	Suggestion for Nepal
Cost-plus pricing	Cost-plus pricing has not been widely used for setting medicine prices.	The most challenging part of cost-plus pricing is obtaining and validating information on different cost components. Clarity and transparency on costs is needed. Cost-plus pricing requires significant technical and human resources.	Using twice the cost of production is suggested.	Apply at the ex-factory or ex-wholesaler levels.	Yes
External price referencing (EPR)	EPR is commonly used for new medicines.	Reference prices have accounted for all forms of discounts, rebates, and taxes with a high degree of confidence, and methods for determining prices follow a transparent and consistent process. EPR requires highly skilled staff to select, collect, check, calculate, and adjust prices over time.	Reference countries: India, Bangladesh, Sri Lanka	It is recommended to apply EPR at the level of the ex-factory price level.	Yes
Internal reference pricing	Internal reference pricing compares and links the prices of medicines that are therapeutically similar and can be substituted for one another within a country. Varies from one country to another. In Brazil, generic medicine price cannot exceed 65% of the respective reference medicine. (Koduah et al., 2022) In the Czech Republic, the first generic medicine has to be priced 32% below the originator, whereas the price of the first biosimilar has to be 15% lower than the reference product. (Vogler & Schneider, 2019)	Internal reference pricing requires technical expertise to determine whether medicines are therapeutically similar.	In Nepal, the first generic medicine should be priced 60% below the originator, whereas the price of the first biosimilar should be only 50% lower than the reference product. For the new presentation in the same dosage of medicine already marketed, the price should be worked out based on the arithmetic mean of the prices of previously launched medicines. (External reference pricing) For the presentation of different dosages of medicines already marketed, the price must not exceed the average price, weighted by sales, of available presentations of medicines that have the same active ingredients, strength, and dosage form.	Internal reference pricing requires resources to maintain a price database and track price variations due to increased competition. Consistent and transparent criteria for pricing generic and biosimilar medicines are explicitly evaluated and stated based on an established methodology. Internal reference pricing enables consistent pricing across medicines with the same or similar therapeutic effects.	Yes
Mark-up regulations			Currently, mark-ups of 6%–10% and 16% are applied to the wholesaler and the retailer in Nepal, respectively (to be applied on a case-by-case basis). A regressive mark-up structure, in which the mark-up rate decreases as the price increases (rather than a fixed percentage mark-up for all prices), needs to be developed.		Yes
Value-based pricing	A highly specialised unit is required for implementation.				To be considered in the future

Cost-plus pricing is the practice of considering costs, including research and development, manufacturing, compliance, overhead, and operational expenses, as well as profits, when setting the price of pharmaceutical products (Cost–plus pricing for setting the price of pharmaceutical products). Using the cost-plus pricing regulation requires significant technical and human resources, as obtaining and validating information on different cost components can be challenging (WHO guideline on country pharmaceutical pricing policies).

Based on these challenges, a 2021 WHO Regional Office for South-East Asia (SEARO) report (World Health Organization’s Regional Office for South-East Asia (SEARO)) states that this policy is no longer recommended. Limited evidence exists about the implementation and outcomes of cost-plus pricing. Although Vietnam, China, Sri Lanka, Bangladesh, Iran, and Pakistan have been identified as using cost-plus pricing, how these countries are implementing this policy remains unclear.

In the past, European countries have used a cost-plus pricing policy; however, none do so anymore (Vogler, 2018). For the cost-plus formula to be used in Nepal, all components, including the way it works and how costs are calculated, must be transparent (Cost–plus pricing for setting the price of pharmaceutical products). The cost-plus pricing approach used in India restricts the price of essential medicines to a maximum of twice the cost of their production (Kumar, 2004). The cost-plus method used in Australia grants a profit margin of around 30% on manufacturing costs (Paris & Belloni, 2014).

Cost-plus pricing must be used in combination with other policies.

#### External price referencing

Another policy to consider for Nepal is external price referencing (EPR), which is being used by an increasing number of countries (External reference pricing, 2021). Under EPR, medicine prices are set based on the prices of the same medicines in other countries. EPR is commonly used for new medicines, and evidence suggests that it is likely to reduce the prices of medicines. To implement this approach, reference prices can be obtained from verifiable data sources and must consider all forms of discounts, rebates, and taxes. EPR should be applied at the ex-factory price level (External reference pricing, 2021). Generally, around five countries are used as reference countries, (Vogler et al., 2008) although some experts recommend that 10 countries should be included (Critchley, 2006; Nguyen et al., 2014). Another challenge is determining the level at which prices should be compared and the ‘price date’ in the reference country (e.g. the current price or the price at launch).

Most European countries have used ex-factory prices for comparison, as this approach eliminates the price variation caused by differences in distribution mark-ups (Nguyen et al., 2014). In Poland, both wholesale and pharmacy retail prices are used for comparison (GO G/O BIG, 2006).

Calculating the benchmark price to implement external price referencing can also present a challenge, although one common approach is to take a price average (Vogler et al., 2008). The benchmark price can also be a fixed percentage of the average price in comparator countries (e.g. 85% for patented, locally produced medicines, and 96% for locally produced generics in Slovenia) (Vogler et al., 2008). In some countries, the benchmark is the lowest price, or the average of the three lowest prices, (Vogler et al., 2008) or the average of the three lowest prices plus 10% (OECD, 2008). External reference pricing systems need to be predictable and transparent, including with respect to reference countries, price sources, and pricing procedures (Espin et al., 2011). The scope of external reference pricing is often limited to originator products (Leopold et al., 2012). For example, generic medicines in France are required to be priced at <50% of the off-patent originator price to be listed for reimbursement (OECD, 2008).

Medicine prices are usually initially defined at the ex-factory and importer levels. Regulation may be implemented at subsequent points along the supply chain to minimise excessive mark-ups by wholesalers and retailers (Vogler et al., 2008).

External reference pricing is often used together with other pricing approaches, such as negotiation. It requires highly skilled staff to select, collect, check, calculate, and adjust prices over time (External reference pricing).

When using EPR, similar characteristics of medicines are considered including location, availability, market size, and originating source. Prices are compared at common points along the distribution chain, from manufacturer to wholesaler to retailer (Koduah et al., 2022). Evidence suggests that external reference pricing is likely to reduce the prices of medicines (Vogler, 2019). It is recommended that EPR should be applied at the ex-factory price level (Vogler, 2019).

Nepal can apply external reference pricing by benchmarking against countries such as India, Bangladesh, and Sri Lanka, all of which are South Asian countries with similar socio-economic parameters (Koduah et al., 2022; Vogler, 2019; Vogler & Schneider, 2019). One of the challenges related to EPR is the availability and accuracy of pricing data sources; checking price accuracy is difficult and requires high-level skills. External reference pricing is widely used in many European countries, as well as in high- and middle-income countries in other parts of the world (Brazil, Egypt, Saudi Arabia, Thailand, Turkey, and the United Arab Emirates) (Babar, 2022; Vogler & Schneider, 2019). In Germany, EPR is a supportive policy and is combined with elements of free pricing and value-based pricing (Vogler & Schneider, 2019).

The methodological approach for the calculation of the reference price uses at least three factors – exchange rates, application of weights, and formula – for calculating the reference price. The use of EPR also requires regular price revisions.

#### Internal reference pricing

Internal reference pricing involves linking prices to those of substitutable – e.g, generic, biosimilar, or therapeutically equivalent – medicines (WHO guideline on country pharmaceutical pricing policies). A specific type of internal price referencing is called a generic price link. This policy refers to the practice of setting the price of a generic relative to (usually a certain percent lower than) the originator's medicine price.

Internal reference pricing is especially useful in the absence of demand-side measures to encourage the preferential prescribing and dispensing of multiple-sourced medicines instead of originator brands. In Nepal, a large majority of medicines used are generic medicines, although some innovator brands are used.

As a policy option, generics in Nepal should be priced 60% below the originators, whereas the price of the first biosimilar could be priced at only 50% lower than the reference medicine. For example, in Lebanon, the price to consumers for generics is 30% less than originator brands (Abdel Rida et al., 2019a; Abdel Rida et al., 2019b). However the figures for Nepal are suggested and they can be discussed further. For example, significant pricing reductions have been seen in the case of generic medicines in Austria, (Martikainen et al., 2015) while successful drug switching can be seen for statins in the Netherlands (Glerum et al., 2020).

Internal reference pricing promotes competition among therapeutically similar medicines, and consistent pricing can be achieved among those medicines. In internal reference pricing, groups of medicines are compared to determine whether they are comparable and interchangeable. After establishing their similarities, the prices of those products are compared at a common point along the distribution chain (for example, ex-factory prices could be used). A benchmark price is then set according to evidence of equivalence (e.g. x milligrams of medicine A achieves the same health effects as x milligrams of medicine B) (Internal reference pricing).

#### Mark-up-regulation policies

A mark-up is an additional charge applied to the price of a product to cover overhead costs, distribution charges, and profit. In the context of the pharmaceutical supply chain, mark-up regulation may apply to wholesale and retail prices.

Mark-up regulations should be used in conjunction with other pricing policies; the mark-up structure should be regressive, where the mark-up rate decreases as the price increases (compared to having a fixed percentage mark-up for all prices). Currently, mark-ups of 6%–10% and 16% are applied to the wholesaler and the retailer, respectively, in Nepal. However, for the retailer, it could be raised up to i.e. 20%.

Mark-up regulations are very helpful in setting clear rules and reducing differences in price for the same medicine along the supply chain. Well-structured mark-up regulations may also provide an incentive to supply certain medicines (e.g. generics and low-volume medicines) if the mark-up levels meet cost and profit expectations (Mark–up regulation across the pharmaceutical supply and distribution chain). Doing so could help protect medicine access for certain patients or population groups (e.g. vulnerable groups or populations living in remote areas).

Transparency is needed when setting mark-ups along the supply and distribution chain, including disclosure of any rebates and discounts. Mark-up regulations also need to be regularly reviewed.

#### Value-based pricing

With the increase in high-cost medicines, value-based pricing is something Nepal can consider in the future. Value-based pricing uses Health Technology Assessment, which requires a well-established governance structure and an advanced level of technical support and expertise. It is most-often applied to on-patent or single-sourced medicines and is normally used along with other pricing mechanisms and policies, such as price negotiation, internal and external reference pricing, and policies to promote generic medicines (Value-based pricing).

#### Price transparency

To effectively utilise pharmaceutical pricing policies in Nepal, transparency is vital. Some of the key features of transparency are sharing the net transaction prices of medicines with relevant stakeholders and disclosing medicine prices along the supply chain. Communicating pricing and reimbursement decisions to the public is also important.

Some examples of price transparency strategies include the transparency directive in the European Union and the publication of the purchasing prices of medical supplies in hospitals in Brazil. Others include the single exit price policy in South Africa and the price disclosure policy in Australia. Despite these examples, price transparency is lacking for medicines in many countries, and more work is needed in that regard (Promoting price transparency).

#### Future implications for policy and practice

Nepal needs a medicines fair pricing policy under the current act and provision with framework of fair pricing, revision and fixing MRP. Nepal needs to set up a medicines pricing unit, whether within the MOHP or as an independent or special unit. Setting up and implementing medicines pricing policies is a complex task, and doing so requires appropriate manpower, resources, and infrastructure. Defining which medicines should be included in the price control list is also important. Establishing monitoring and evaluation of medicine prices is an equally vital task; hence, manpower with expertise in medicines, pharmacy, health economics, and pricing regulation is needed. A combination of the above policies could be used.

Nepal is on the way to strengthening its pharmaceutical regulatory capacity, and policies to ensure medicines quality and safety, the quality use of generic medicines, and rational prescribing could go hand in hand with the price control mechanisms. Establishing better profit margins and maintaining them for community pharmacists may help sustain viable businesses and improved patient health outcomes.

One of the major challenges in Nepal is the disparity of profit margins along the medicines supply chain. That disparity has repercussions for access to and affordability of medicines and also has an impact on how the pharmaceutical industry and community pharmacies work and on prescribing behaviour. In this context, price transparency is needed along the supply chain by defining and implementing profit margins. Implementation of pricing policies also requires regulation and strengthening of the workforce. A notice regarding the price of medicine could also be published in the Nepal Gazette.

## Conclusion

Key issues related to the supply chain, profit margins, add-ons, mark-ups, prescribing of medicines, and medicines pricing were identified. In this context, a set of pricing policies were identified and discussed. Enactment of medicines fair pricing rule with a range of policies, including cost-plus pricing, external price referencing, internal reference pricing, and mark-up policies are needed. Price transparency along the supply chain is also needed and can be accomplished by defining and implementing profit margins. Regardless of whether some form of price control is observed in Nepal, the country is missing a coherent policy. Hence, suggestions made in this paper could be generally useful for informing a rational and fair pricing structure and improving access to medicines.

## Appendix 1

List of abbreviations

**Table:**

APPON	Association of Pharmaceutical Producers of Nepal
CR	cost ratio
CV	cost variation
DDA	Department of Drug Administration
EPR	external price referencing
GMP	good manufacturing practices
INN	International Nonproprietary Name
LMIC	low- and middle-income country
MIAN	Medicine Importers Association
MOHP	Ministry of Health and Population
MRP	maximum retail price
MTaPS	Medicines, Technologies, and Pharmaceutical Services
NCD	non-communicable diseases
NCDA	Nepal Chemist and Druggist Association
PDAN	Pharmaceutical Distributers Association of Nepal
R&D	research and development
SAARC	South Asian Association for Regional Cooperation
WHO	World Health Organization
